# Machine Learning-Based Plasma Metabolomics in Liraglutide-Treated Type 2 Diabetes Mellitus Patients and Diet-Induced Obese Mice

**DOI:** 10.3390/metabo14090483

**Published:** 2024-09-02

**Authors:** Seokjae Park, Eun-Kyoung Kim

**Affiliations:** 1 Department of Brain Sciences, Daegu Gyeongbuk Institute of Science and Technology, Daegu 42988, Republic of Korea; godclover7@dgist.ac.kr; 2 Neurometabolomics Research Center, Daegu Gyeongbuk Institute of Science and Technology, Daegu 42988, Republic of Korea

**Keywords:** type 2 diabetes mellitus, obesity, liraglutide, metabolomics, metabolic profiling, machine learning

## Abstract

Liraglutide, a glucagon-like peptide-1 receptor agonist, is effective in the treatment of type 2 diabetes mellitus (T2DM) and obesity. Despite its benefits, including improved glycemic control and weight loss, the common metabolic changes induced by liraglutide and correlations between those in rodents and humans remain unknown. Here, we used advanced machine learning techniques to analyze the plasma metabolomic data in diet-induced obese (DIO) mice and patients with T2DM treated with liraglutide. Among the machine learning models, Support Vector Machine was the most suitable for DIO mice, and Gradient Boosting was the most suitable for patients with T2DM. Through the cross-evaluation of machine learning models, we found that liraglutide promotes metabolic shifts and interspecies correlations in these shifts between DIO mice and patients with T2DM. Our comparative analysis helped identify metabolic correlations influenced by liraglutide between humans and rodents and may guide future therapeutic strategies for T2DM and obesity.

## 1. Introduction

Type 2 diabetes mellitus (T2DM) and obesity are major global health issues that have significant complications and increase the risk of morbidity and mortality [1,2]. Over the past 20 years, therapies based on incretin hormones, particularly glucagon-like peptide-1 receptor agonists (GLP-1 RAs), have become the preferred treatment for T2DM and obesity due to their efficacy and safety in numerous clinical trials [2,3,4]. Among the most widely adopted GLP-1 RAs, liraglutide substantially improves glycemic control and promotes weight loss [5,6,7,8,9,10]. Recent advancements in omics technologies, including proteomics and metabolomics, have demonstrated that liraglutide treatment significantly changes protein expression and metabolite levels across various biological samples in patients with T2DM and obesity and in mouse models [11,12,13,14,15,16,17]. The identified proteins and metabolites provide insights into liraglutide’s multifaceted mechanisms of action, which improve glycemic control and lipid profiles, reduce inflammation, and potentially offer cardiovascular and renal benefits [11,12,13,14,15,16,17].

Metabolomics encompasses the analysis of a vast array of metabolites present in biological samples using sophisticated analytical techniques. The ongoing challenges of such analysis include obtaining metabolic snapshots and surrogate diagnostics, identifying biomarkers, elucidating the mechanisms driving metabolic disorders, and evaluating drug efficacy and therapeutic outcomes [18,19]. Understanding the metabolic shifts induced by liraglutide treatment can provide valuable insights into its mechanisms of action and help identify potential biomarkers for treatment response. Previous metabolomic studies using different analytical platforms in rodents and humans demonstrated that liraglutide induced various metabolic shifts in multiple tissues and blood. For example, liraglutide coordinates the modulation of fatty acid metabolism in the hypothalamus and skeletal muscle as well as amino acid and carbohydrate metabolism in the plasma and liver in diet-induced obese (DIO) mice [11]. In the urine of DIO mice, significant alterations were observed in nicotinamide adenine dinucleotide metabolism and the β-oxidation of fatty acids following liraglutide treatment [12]. Human plasma and serum metabolomics studies have demonstrated remarkable changes in ketone bodies, citrate, branched-chain amino acids, and glycerophospholipids after liraglutide treatment [13,14]. Although these metabolic changes reflect the metabolic effects of liraglutide, no study has identified or integrated common metabolites altered by liraglutide between rodents and humans due to the lack of comparable datasets that match experimental parameters such as biological sample types and data processing methods. Therefore, a comparative metabolomics approach, bridging preclinical and clinical studies of liraglutide treatment, is needed to fully understand the metabolic changes that occur in rodents and humans. Also, identifying and targeting key metabolism or metabolic pathways altered by liraglutide may help develop advanced therapeutic strategies that enhance liraglutide efficacy for personalized medicine approaches for T2DM and obesity treatment.

Machine learning uses artificial intelligence methodologies to automatically analyze large-scale and complex data, and its use is steadily growing across all fields of biomedical science [20,21,22,23]. Machine learning is becoming increasingly integrated into metabolomics, enhancing the accuracy of data analysis and consolidating various types of omics data to provide a comprehensive view of biological systems. Consequently, machine learning is revolutionizing metabolomics by enabling the analysis of complex datasets, improving disease diagnosis, predicting clinical outcomes, and facilitating biomarker discovery [24,25,26,27]. The application of machine learning in metabolomic studies enables a thorough, comprehensive understanding of the metabolic efficacy of liraglutide. This approach not only aids the discovery of novel biomarkers for the outcomes of GLP-1RA intervention but also advances the development of personalized therapeutic strategies, making it a critical tool in translational research.

Here, we report machine learning-based metabolomic analysis aimed at understanding the metabolic effects of liraglutide using the plasma metabolomes of DIO mice and patients with T2DM. This comprehensive and comparative analysis will offer new insights that could lead to more precise and personalized interventions for T2DM and obesity.

## 2. Materials and Methods

### 2.1. Data Collection

The metabolomics analysis was based on the plasma metabolome raw dataset obtained from post-liraglutide treatment studies, including our previous study (liraglutide treatment in male DIO mice fed a high-fat diet) [11] and the open-source data from the Metabolomics Workbench of the National Institutes of Health (liraglutide treatment in male patients with T2DM; https://www.metabolomicsworkbench.org, Project ID PR001699, Project DOI: 10.21228/M83B08, accessed on 2 July 2023). The Metabolomics Workbench is supported by grants #U2C-DK119886, #3OT2OD030544, and #1U2C-DK119889.

Among the DIO mice, one group received vehicle treatment (Veh, *n* = 8), and the other received liraglutide treatment (Lira, *n* = 8). For the patients with T2DM, the datasets consisted of samples collected before (Pre, *n* = 20) and after (Post, *n* = 20) liraglutide treatment. These metabolomic data were obtained from 20 distinct patients before and after liraglutide treatment. The plasma samples from DIO mice and patients with T2DM were analyzed using gas chromatography–tandem mass spectrometry (GC-MS/MS, Agilent Technologies, Santa Clara, CA, USA) for targeted and untargeted analysis and liquid chromatography quadrupole time-of-flight mass spectrometry (LC-QTOF-MS, Waters Corp., Milford, MA, USA) for untargeted analysis, respectively.

### 2.2. Data Processing

The raw data were converted to analysis base files (“.abf”) using ABF converter (Reifycs Inc., Tokyo, Japan), and metabolic features (peak detection, deconvolution, identification, and alignment) were processed using MS-DIAL v5.2.240424.3 software (RIKEN, Waco, Japan) [28] with the following parameters: mass scan range, 0 to 2000 Da; mass tolerance for centroid, 0.01 Da; retention time tolerance for alignment, 0.1 min; spectrum similarity tolerance for alignment, 0.8 min; mass tolerance for alignment, 0.5 Da; identification, FiehnRI and ESI(±)-MS/MS standard database. The partial least squares discriminant analysis (PLS-DA) was performed, and volcano plots were generated in the Metaboanalyst 6.0 online tool [29]. To identify and remove variables, data were filtered and normalized with the following parameters: variance filter, interquartile range; abundance filter, mean intensity value; sample normalization, median; data transformation, log (base 10); data scaling, auto. After data processing, Kyoto Encyclopedia of Genes and Genomes (KEGG)-based metabolite set enrichment analysis and pathway topological analysis were performed using default values in Metaboanalyst 6.0 [30].

### 2.3. Machine Learning Processing

Machine learning was performed using Orange v3.37.0 software based on Python, an open-source data visualization and analysis tool for data mining through visual programming (University of Ljubljana, Ljubljana, Slovenia) [31]. The datasets were preprocessed using the ‘Preprocess’ widget. Rows with missing values were removed, and 80% of the data were randomly selected. To prepare the data for model training, all numerical features were standardized to have a range of −1 to 1.

This standardization allowed seven machine learning algorithms to be implemented to build classification models, each configured with specific parameters to optimize their performance [21,32]. Logistic regression used the least absolute shrinkage and selection operator algorithm with the L1 constraint set to 0.01 to enforce sparsity in the model. For Random Forest, 5000 trees were used to ensure replicable training and robust performance. A Neural Network was implemented with the rectified linear unit activation function, and over 2000 iterations were used to train the model to capture non-linear relationships in the data. The k-Nearest Neighbors (kNN) algorithm used Euclidean distances to measure the proximity of instances. Adaptive Boost (AdaBoost) was configured with 2000 estimators and a learning rate of 0.5, employing the stagewise additive modeling using a multi-class exponential real algorithm to improve classification accuracy by focusing on misclassified instances. Gradient Boosting, which used the eXtreme Gradient Boosting (XgBoost) algorithm, was set up with 5000 trees, a learning rate of 0.7, and a lambda regularization parameter of 1 to control overfitting and enhanced model generalization. The Support Vector Machine (SVM) classifier was configured with a cost parameter of 1, a regression loss epsilon of 0.1, a radial basis function kernel, and 2000 iterations to handle the classification task effectively by maximizing the margin between classes.

The data were imported using the ‘File’ widget, and preprocessing tasks were handled by the ‘Preprocess’ widget. The ‘Test & Score’ widget was used for model training and evaluation, enabling the systematic comparison of model performance. The ‘Confusion Matrix’ and ‘Receiver operating characteristic (ROC) Analysis’ widgets provided a detailed performance evaluation of each classifier. The datasets were subjected to cross-validation using Monte Carlo methods, ensuring the robustness and reliability of the results. The datasets were randomly divided into training and test sets, with 75% of the data used for training and 25% reserved for testing.

Model performance was assessed using multiple metrics, including the mean values of classification accuracy, precision, recall, F1 score, area under the ROC curve (AUC), and the Matthews correlation coefficient score [33]. These metrics comprehensively evaluate each model’s predictive capability and generalization performance.

To learn the significance of each feature in our classification models, we employed feature scoring (feature importance analysis) using the ‘Rank’ widget. Specifically, we utilized the feature importance analysis metrics, including Information Gain, Gini Index, Chi-squared, ReliefF, and ANOVA, to quantify the importance of individual features.

The normalized peak intensities were converted to *z*-scores to represent the data points on a standard scale for data visualization. The *z*-score was calculated using the formula: *z* = *X* − *μ*/*σ*, where *X* presents the raw value of peak intensity, *μ* is the mean of the population, and *σ* is the standard deviation of the population.

Machine learning was conducted on a computer equipped with an Intel Core i7 processor and 32 GB of RAM running on Windows 11. This setup ensured that computational resources were sufficient to efficiently handle the data processing and model training tasks.

### 2.4. Statistical Analysis

In the Supplementary Figures, the quantitative data are presented as the mean ± standard error of the mean (SEM). To compare the two groups, datasets were analyzed by a two-tailed unpaired Student’s *t*-tests. Multiple comparisons were performed by two-way ANOVA followed by Fisher’s LSD test using GraphPad Prism software (v10.2.3; Dotmatics, Boston, MA, USA). A value of *p* < 0.05 was considered to indicate a significant difference.

## 3. Results

### 3.1. Study Design

**Figure 1:**
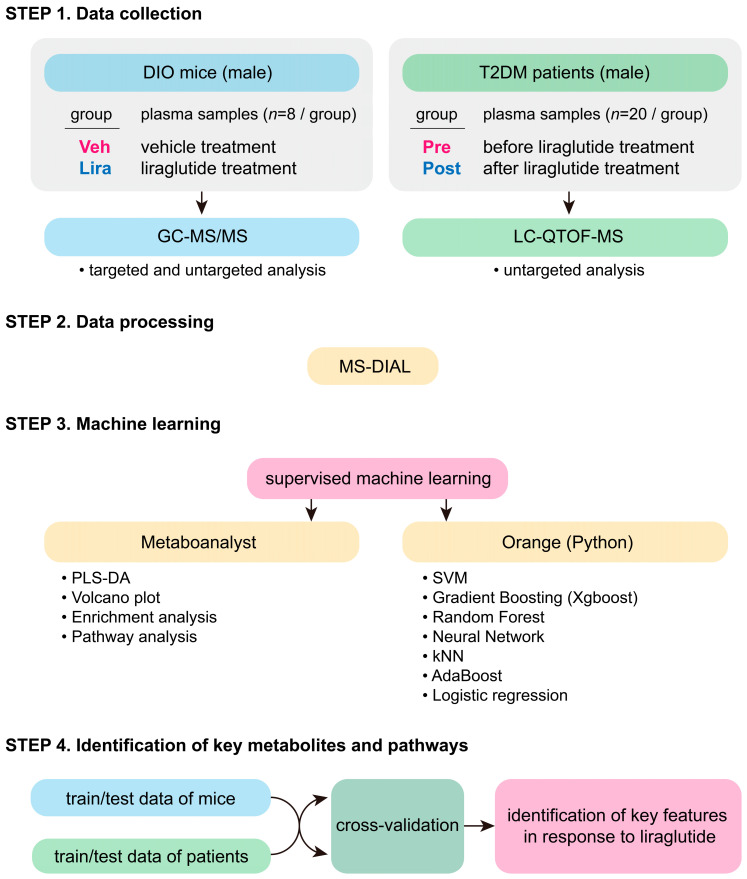
Study design and data processing workflow.

The overall workflow of this study and detailed information on each type of analysis of the DIO mice and patients with T2DM are presented in Figure 1.

The datasets used in this study were derived from our previous study [11] or were open-source data from the Metabolomics Workbench of the National Institutes of Health. After data collection, the raw datasets were processed using MS-DIAL, which involved peak detection, deconvolution, identification, and alignment. Subsequently, supervised machine learning models were used with model training, parameter optimization, and performance evaluation. The machine learning processes used various tools, including Metaboanalyst for simple metabolomic profiling and Orange (Python) for machine learning tasks. Finally, through the cross-evaluation of model performance, we identified common key metabolites and metabolic pathways changed by liraglutide in both humans and mice (Figure 1).

### 3.2. Changes in Plasma Metabolome in DIO Mice in Response to Liraglutide

**Figure 2:**
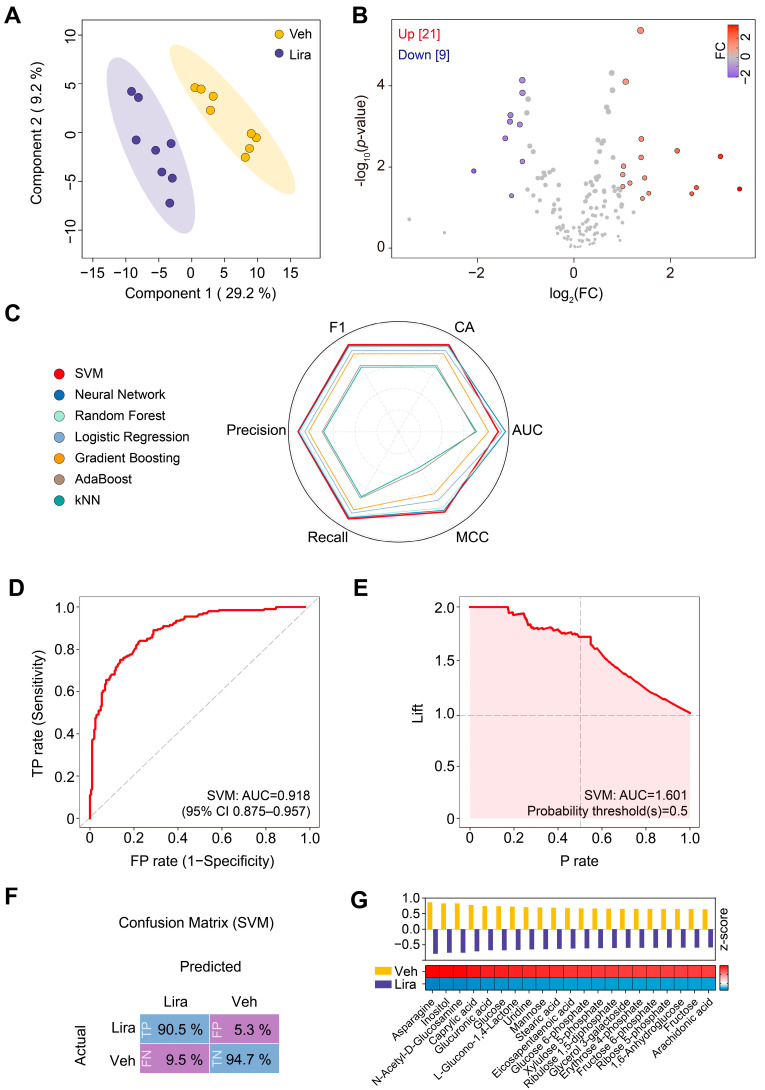
Changes in plasma metabolites in DIO mice in response to liraglutide. (**A**) Partial least squares discriminant analysis (PLS-DA) plot. (**B**) Volcano plot. FC, fold change. (**C**) Radar chart comparing the performance of seven machine learning models. CA, classification accuracy. AUC, area under the receiver operating characteristic (ROC) curve. MCC, Matthews correlation coefficient. F1, 2 × (Precision × Recall/Precision + Recall). (**D**) ROC curve of the SVM model. TP rate, true positive rate; FP rate, false positive rate. The calculation of the 95% confidence interval (CI) was based on the mean and covariance of 100 random sampling tests. (**E**) Performance lift curve of the SVM model. Lift, predicted positive event rate; P rate, precision rate. (**F**) Confusion matrix for the SVM model. TN, true negative; FN, false negative. Veh, vehicle; Lira, liraglutide treatment. (**G**) Top-ranked metabolic shifts. The bar graph and the heatmap were generated using the *z*-score of normalized peak intensity.

To characterize the effects of liraglutide on the plasma metabolome in DIO mice, we performed a metabolomic analysis to compare the liraglutide- and vehicle-injected groups. Notably, PLS-DA distinguished the two groups, indicating that the metabolome of DIO mice is remodeled by liraglutide (Figure 2A). In total, 30 annotated metabolites showed statistically significant differences between the groups (Figure 2B).

To determine the best-performing machine learning model for identifying potential vital metabolites, we evaluated the performance of SVM, Neural Network, Random Forest, Logistic Regression, Gradient Boosting, AdaBoost, and kNN on the mouse plasma metabolome. Among these algorithms, we chose the SVM owing to its higher performance metrics (Figure 2C and Supplementary Table S1). The SVM model yielded a high AUC score of 0.918 (95% confidence interval [CI]: 0.875–0.957) (Figure 2D). We generated a performance lift curve for the SVM model to validate its robustness and potential utility in metabolomics analysis. The curve showed that the SVM model had high prediction accuracy, especially at the positive classification thresholds (probability thresholds ≥ 0.5), indicating strong performance in identifying key metabolites (Figure 2E). To further evaluate the performance of our SVM model, we analyzed the confusion matrix. This analysis revealed that the model had a high percentage of true positives (90.5%) and true negatives (94.7%), indicating solid predictive capabilities (Figure 2F).

By applying our SVM model, we identified the top-ranked metabolites that were most likely the critical contributors to the observed metabolic changes. These metabolites were selected on the basis of their significant features generated by the model (Figure 2G). Liraglutide, as predicted by the SVM model, significantly decreased the levels of metabolites such as asparagine, inositol, N-acetyl-D-glucosamine, caprylic acid, glucuronic acid, glucose, L-glucono-1,4-lactone, uridine, mannose, stearic acid, eicosapentaenoic acid, glucose 6-phosphate, xylulose 5-phosphate, ribulose 1,5-diphosphate, glycerol 3-galactoside, erythrose 4-phosphate, fructose 6-phosphate, ribose 5-phosphate, 1,6-anhydroglucose, fructose, and arachidonic acid in DIO mice compared with the vehicle group (Figure 2G).

### 3.3. Changes in Plasma Metabolome in Patients with T2DM in Response to Liraglutide

**Figure 3:**
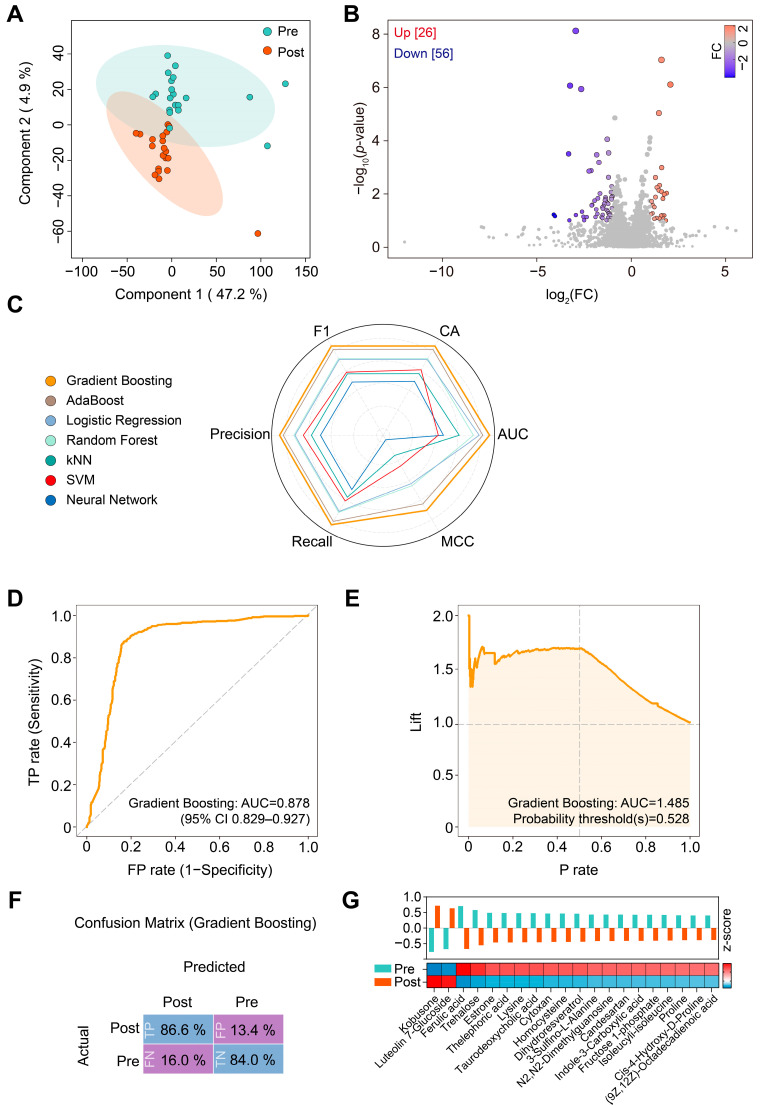
Changes in plasma metabolites in patients with T2DM in response to liraglutide. (**A**) PLS-DA plot. (**B**) Volcano plot. FC, fold change. (**C**) Radar chart comparing the performance of seven machine learning models. (**D**) ROC curve of the Gradient Boosting (XgBoost) model. The calculation of the 95% confidence interval (CI) was based on the mean and covariance of 100 random sampling tests. (**E**) Performance lift curve of the Gradient Boosting model. Lift, predicted positive event rate; P rate, precision rate. (**F**) Confusion matrix for the Gradient Boosting model. Pre, pre-liraglutide treatment; Post, post-liraglutide (Post) treatment. (**G**) Top-ranked metabolic shifts. The bar graph and the heatmap were generated using the *z*-score of normalized peak intensity.

To characterize the effects of liraglutide on the plasma metabolome in patients with T2DM, we performed metabolomic analysis to compare the pre-liraglutide treatment group to the post-liraglutide one. PLS-DA distinguished these two groups, indicating that the metabolome of patients with T2DM was remodeled by liraglutide (Figure 3A). In total, 82 annotated metabolites showed statistically significant differences between the groups (Figure 3B).

We evaluated the performance of different machine learning algorithms on the plasma metabolome of the patients with T2DM using Gradient Boosting, AdaBoost, Logistic Regression, Random Forest, kNN, SVM, and Neural Network. We chose Gradient Boosting owing to its markedly higher performance metrics than those of the other algorithms (Figure 3C and Supplementary Table S2). The Gradient Boosting model yielded a high AUC score of 0.878 (95% CI: 0.829–0.927) (Figure 3D). Next, we generated a performance lift curve for this model, which indicated strong performance in identifying key metabolites at the positive classification thresholds (probability thresholds ≥ 0.5) (Figure 3E). To further evaluate the performance of our Gradient Boosting model, we analyzed the confusion matrix. This analysis revealed that the model had a high percentage of true positives (86.6%) and true negatives (84.0%), indicating solid predictive capabilities (Figure 3F).

By applying our Gradient Boosting model, we identified the top-ranked metabolites that were most likely the key contributors to the observed metabolic changes (Figure 3G). Post-liraglutide treatment, as predicted by the Gradient Boosting model, significantly decreased the levels of metabolites, such as ferulic acid, trehalose, estrone, lysine, taurodeoxycholic acid, homocysteine, 3-sulfino-L-alanine, indole-3-carboxylic acid, fructose 1-phosphate, isoleucyl-isoleucine, proline, *cis*-4-hydroxy-D-proline, and (9Z, 12Z)-octadecadienoic acid (linoleic acid), compared with the pre-liraglutide group (Figure 3G).

### 3.4. Changes in Plasma Metabolites and Metabolic Pathways Following Liraglutide Treatment in DIO Mice and Patients with T2DM

**Figure 4:**
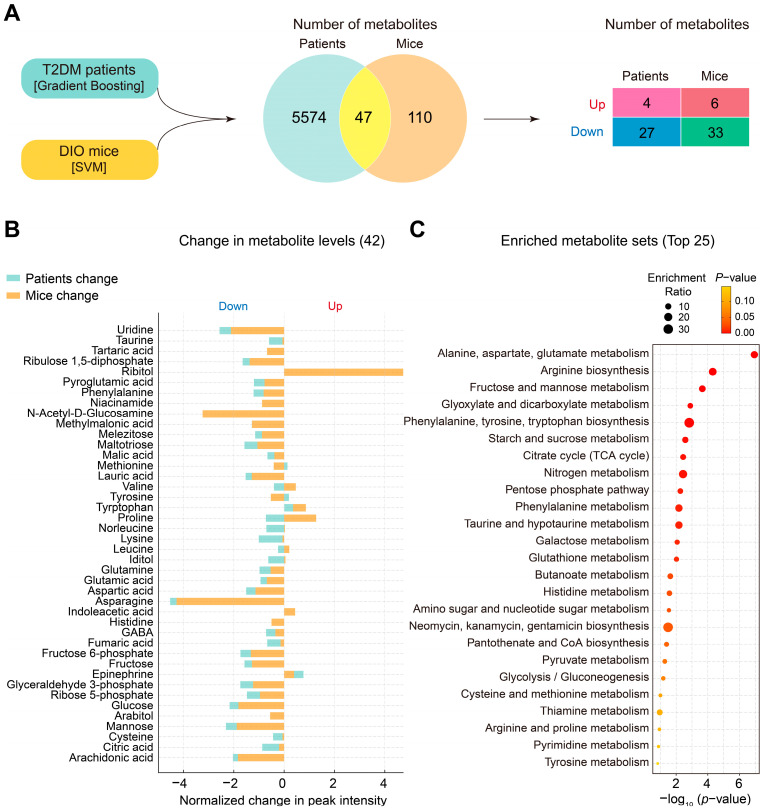
Comparison of plasma metabolomic changes between DIO mice and patients with T2DM. (**A**) A Venn diagram showing the overlapping and unique metabolites identified in patients with T2DM and DIO mice after processing with Gradient Boosting and SVM, respectively. (**B**) Changes in metabolite levels. In the bidirectional bar graph, the left side represents decreased, and the right side represents increased. The length of the bars indicates the extent of the increase or decrease. (**C**) Top-ranked metabolite set enrichment analysis. Circle size indicates the expected enrichment value for a metabolic pathway.

To understand the metabolic similarities and differences in response to liraglutide between DIO mice and human patients with T2DM, we compared the plasma metabolomic data between the two species. We used data from the Gradient Boosting model for patients with T2DM and the SVM model for DIO mice and identified 47 metabolites overlapping between them (Figure 4A). We categorized the common metabolites into increased and decreased. Four overlapping metabolites were increased and 27 were decreased in patients with T2DM, whereas 6 metabolites were increased and 33 were decreased in DIO mice (Figure 4A).

Among the overlapping metabolites, 42 showed changes in levels due to liraglutide treatment. Two metabolites (tryptophan and epinephrine) increased, while 26 metabolites (uridine, taurine, ribulose 1,5-diphosphate, pyroglutamic acid, phenylalanine, methylmalonic acid, melezitose, maltotriose, malic acid, lauric acid, lysine, glutamine, glutamic acid, aspartic acid, asparagine, GABA, fumaric acid, fructose 6-phosphate, fructose, glyceraldehyde 3-phosphate, ribose 5-phosphate, glucose, mannose, cysteine, citric acid, and arachidonic acid) decreased in both DIO mice and patients with T2DM. Seven metabolites (methionine, valine, tyrosine, proline, norleucine, leucine, and iditol) changed in opposite directions. In DIO mice only, tartaric acid, niacinamide, histidine, and arabitol were decreased, while ribitol, N-acetyl-D-glucosamine, and indoleacetic acid were increased by liraglutide (Figure 4B).

To identify the metabolic pathways impacted by liraglutide in both DIO mice and patients with T2DM, we performed a metabolite set enrichment analysis for the 42 overlapping metabolites. The most pronounced impacts of liraglutide were on metabolites involved in alanine, aspartate, and glutamate metabolism; arginine biosynthesis; fructose and mannose metabolism; glyoxylate and dicarboxylate metabolism; phenylalanine, tyrosine, and tryptophan biosynthesis; starch and sucrose metabolism; the citric acid cycle; nitrogen metabolism; the pentose phosphate metabolism; and taurine and hypotaurine metabolism (Figure 4C).

### 3.5. Identification of Key Plasma Metabolites and Metabolic Pathways Changed by Liraglutide in Both DIO Mice and Patients with T2DM 

**Figure 5:**
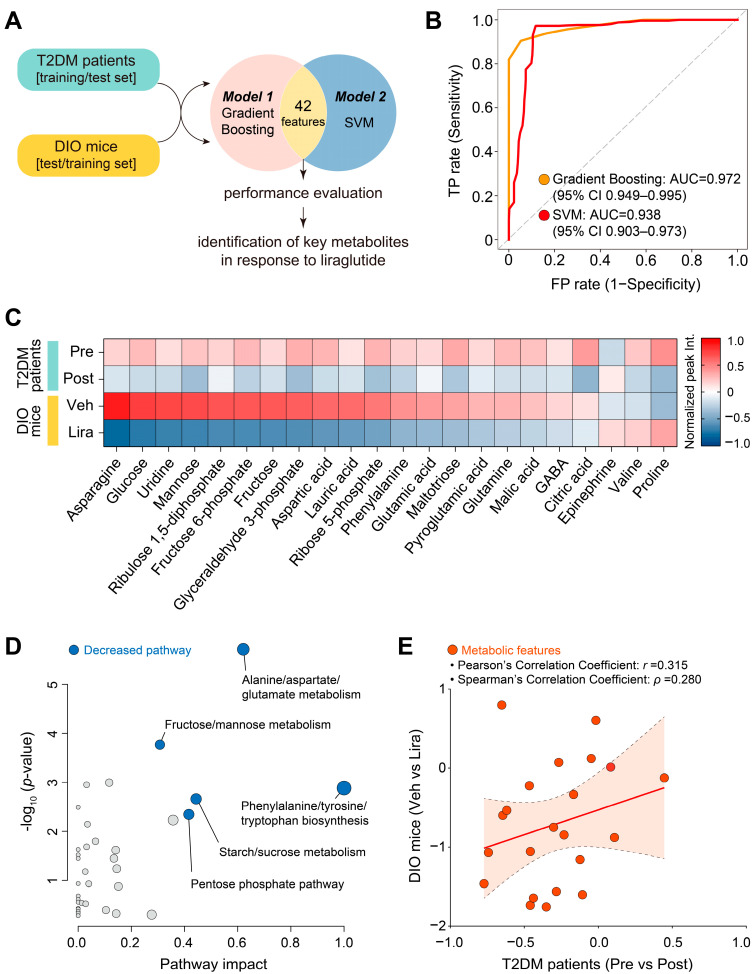
Cross-evaluation and correlation analyses between DIO mice and patients with T2DM. (**A**) Study design for cross-evaluation. (**B**) ROC curves of the Gradient Boosting and SVM models. The calculation of the 95% confidence interval (CI) was based on the mean and covariance of 100 random sampling tests. (**C**) Heatmap of metabolites significantly altered by liraglutide that were common to DIO mice and patients with T2DM. Color gradient represents normalized peak intensity. (**D**) Topological analysis of metabolic pathways. Blue dots represent pathways decreased by liraglutide. (**E**) Correlation plot of metabolic responses to liraglutide in DIO mice and patients with T2DM. The *X*- and *Y*-axes represent metabolic changes induced by liraglutide from patients with T2DM and DIO mice, respectively. The reddish-orange dots indicate individual metabolic features. The red line shows R-squared (*R*^2^), and its value is 0.064 (95% CI: −0.189–0.609). Pearson’s (*r*) and Spearman’s (*ρ*) correlation coefficients are shown above the graph. The criteria for Pearson’s correlation coefficients were as follows: <0.1, no correlation; 0.1 to <0.3, weak correlation; 0.3 to <0.5, moderate correlation; 0.5 to <0.7, strong correlation; 0.7 to 1.00 very strong correlation. The criteria for Spearman’s correlation coefficients were as follows: <0.1, no correlation; 0.1 to <0.4, weak correlation; 0.4 to <0.8, moderate correlation; 0.8 to 1.0, strong correlation.

To identify key plasma metabolites and metabolic pathways changed in both DIO mice and patients with T2DM, we conducted a performance cross-evaluation of Gradient Boosting and SVM. We used the 42 overlapping metabolites from DIO mice as a training dataset for SVM and those from patients with T2DM as a test dataset for Gradient Boosting, and vice versa (Figure 5A). The ROC curves for Gradient Boosting and SVM showed high AUC scores of 0.972 (95% CI: 0.949–0.995) and 0.936 (95% CI: 0.903–0.973), respectively. The performance of Gradient Boosting was superior to that of SVM, suggesting that a well-tuned and optimized Gradient Boosting model is more suitable for identifying and analyzing key liraglutide-responsive metabolites and metabolic pathways common to DIO mice and patients with T2DM (Figure 5B).

Out of the 42 overlapping metabolites, we identified 22 that were statistically significant in both DIO mice and patients with T2DM. Asparagine, glucose, uridine, mannose, ribulose 1,5-diphosphate, fructose 6-phosphate, fructose, glyceraldehyde 3-phosphate, aspartic acid, lauric acid, ribose 5-phosphate, phenylalanine, glutamic acid, maltotriose, pyroglutamic acid, glutamine, malic acid, GABA, and citric acid were significantly decreased by liraglutide in both DIO mice and patients with T2DM (Figure 5C and Supplementary Figure S1). By contrast, the levels of epinephrine were increased by liraglutide. Notably, the levels of valine and proline were remarkably decreased by liraglutide in patients with T2DM but were increased in DIO mice (Figure 5C and Supplementary Figure S1).

The common 19 decreased metabolites were associated with metabolic pathways such as phenylalanine, tyrosine, and tryptophan biosynthesis; alanine, aspartate, and glutamate metabolism; fructose and mannose metabolism; starch and sucrose metabolism, and the pentose phosphate pathway (Figure 5D).

To further analyze the metabolic proximity and relationships between significantly changed metabolites in DIO mice and patients with T2DM treated with liraglutide, we generated a cross-species correlation plot using various statistics, including linear measures (R-squared and Pearson’s correlation coefficient) and a non-linear measure (Spearman’s correlation coefficient). Correlation analysis showed a moderate positive linear and rank correlation between changes in metabolite levels in mice and humans after liraglutide treatment, indicating that both species exhibit similar patterns in their metabolite responses to liraglutide, at least for these common metabolites (Figure 5E).

Overall, liraglutide triggered similar plasma metabolome changes (20 metabolites, with valine and proline showing opposite changes) in both species. This finding suggests the critical metabolites involved in the response to liraglutide and provides valuable insights into its mechanism of action and response efficacy. Our finding will facilitate the advancement of translational research employing liraglutide intervention for the treatment of metabolic diseases such as T2DM and obesity.

## 4. Discussion

Numerous metabolic signatures associated with liraglutide treatment have been identified in either clinical or preclinical practice to understand metabolic changes in plasma, serum, urine, and various tissues [11,12,13,14,34]. Despite these efforts, interpreting sophisticated metabolomics data has been challenging, necessitating the development of new analytical methods. In this study, using metabolomics analysis combined with machine learning approaches, we found that liraglutide caused several common metabolic shifts in DIO mice and patients with T2DM. Twenty metabolites were identified as key indicators of the metabolic response to liraglutide treatment in both species; these metabolites had similar patterns in a cross-species correlation plot. Our study revealed the unique advantages of applying machine learning-based metabolomics to facilitate the understanding of liraglutide’s efficacy in terms of metabolic changes in both rodents and humans, thereby providing clinical translation potential in the future.

Obesity and insulin resistance are often associated with elevated plasma levels of amino acids, particularly alanine, glutamic acid, and phenylalanine. The elevated plasma amino acids promote oxidative stress and inflammation, increasing cardiovascular risk and promoting non-alcoholic fatty liver disease [35,36,37]. In our study, metabolomic machine learning data showed that liraglutide significantly reduced the plasma levels of phenylalanine and glutamic acid, which may help lower inflammatory responses and improve insulin sensitivity [38]. During insulin resistance, impaired mitochondrial respiration increases the metabolic fluxes of gluconeogenesis and the citric acid cycle, which subsequently leads to oxidative stress and recruitment of pro-inflammatory cytokines, such as tumor necrosis factor-α (TNF-α) and interleukin 6 [39]. Our machine learning results showed that the intermediates of gluconeogenesis and the citric acid cycle, such as glucose, fructose 6-phosphate, glyceraldehyde 3-phosphate, malic acid, and citric acid, were significantly decreased by liraglutide in both DIO mice and patients with T2DM, which may also contribute to increasing insulin sensitivity by mitigating pro-inflammatory responses and excessive oxidative flux in obesity and T2DM [38,40,41].

Our study demonstrated an increase in plasma epinephrine levels following liraglutide treatment in both DIO mice and patients with T2DM. This increase can elevate metabolic rate and suppress appetite, leading to weight loss, which is consistent with the effects of liraglutide [42,43]. However, liraglutide may trigger a rapid lowering of blood glucose, which could activate the sympathetic nervous system and the subsequent release of epinephrine to prevent hypoglycemia, likely as a compensatory response [44,45,46]. Therefore, our study suggests a potential mechanism by which liraglutide regulates blood glucose by modulating epinephrine levels. In terms of the potential implications for patient care, the elevated plasma epinephrine levels also raise concerns in patients with T2DM regarding the risk of hypoglycemia, as they are more vulnerable to fluctuations in blood glucose levels [47,48]. This may require careful monitoring and individualized treatment plans when using liraglutide in clinical practice. Future studies should further explore these implications to optimize the safety and efficacy of liraglutide therapy in managing T2DM and obesity.

Interestingly, our metabolomic analysis showed that liraglutide markedly reduced ribose 5-phosphate in both DIO mice and patients with T2DM while also decreasing NADPH in patients with T2DM (Supplementary Figures S1 and S2). The pentose phosphate pathway (PPP) is critical in glycemic control, producing NADPH for cellular redox balance and ribose 5-phosphate for nucleotide and nucleic acid biosynthesis [49]. In obesity and T2DM, free fatty acids released from adipocytes promote the PPP flux in M1 macrophages, leading to excessive NADPH production [49,50]. Excess NADPH induces oxidative stress and inflammatory responses by promoting the secretion of pro-inflammatory cytokines, such as TNF-α and interleukin 1β, and leads to severe insulin resistance in various tissues [49,50]. Notably, liraglutide reduces the production of these cytokines [51,52]. As suggested by our metabolomic analysis, liraglutide-reduced PPP flux may prevent pro-inflammatory responses and improve insulin sensitivity.

Integrating machine learning into metabolomics study is a powerful tool that complements and extends traditional statistical methodologies [25]. By enabling the efficient handling of large-scale data, detecting nonlinear relationships, automating feature selection, facilitating personalized analysis, and uncovering unexpected discoveries, machine learning allows for a deeper and more comprehensive understanding of metabolomic data. Compared to conventional methods, machine learning empowers researchers to explore the complexity and multidimensionality of metabolomic datasets more effectively, ultimately enhancing our understanding of biological processes and contributing significantly to elucidating disease mechanisms and developing therapeutic strategies.

Several limitations of our study should be noted. Firstly, differences in mass spectrometry platforms can significantly influence the number of annotated metabolites identified, as shown in the Venn diagram of Figure 4A. LC analysis yields a substantially higher number of identified metabolites compared with GC. Consequently, it is imperative to conduct additional analyses using LC to enhance the detection of metabolites in DIO mice. Additionally, the post-treatment schemes were not matched. The vehicle- and liraglutide-injected DIO mice were from separate groups, whereas for patients with T2DM, the comparison was made within the same group before and after liraglutide treatment. A possible alternative could be comparing the DIO mice before and after liraglutide treatment or including a placebo control group for patients with T2DM. Finally, this study did not account for inherent differences between rodents and humans, such as race (strain), age, sex, and metabolic characteristics, which could cause variability in the observed metabolic profiles. Therefore, a cautious interpretation of the findings is needed, and further clinical or preclinical research is warranted to validate and optimize the results.

## 5. Conclusions

In conclusion, using machine learning techniques, our study revealed numerous common metabolic shifts induced by liraglutide treatment in DIO mice and patients with T2DM. Our findings can be integrated into medical or pharmacological applications to reveal treatment outcomes and develop new therapeutic strategies. The framework highlights the unique aspects of machine learning-based interpretation of metabolomics data for clinical diagnostics and prognostics through GLP-1 RA interventions.

## Data Availability

The original contributions presented in the study are included in the article and Supplementary Materials, further inquiries can be directed to the corresponding author.

## Supplementary Materials

The following supporting information can be downloaded at: https://www.mdpi.com/article/10.3390/metabo14090483/s1, Figure S1, Plasma metabolites altered by liraglutide in patients with T2DM and DIO mice; Figure S2, Effect of liraglutide on plasma NADPH level in patients with T2DM; Table S1, Model performance metrics in DIO mice; Table S2, Model performance metrics in patients with T2DM.
