# Supplementary material for: Machine Learning-Based Plasma Metabolomics in Liraglutide-Treated Type 2 Diabetes Mellitus Patients and Diet-Induced Obese Mice
Source: Metabolites. 2024 Sep 2;14(9):483. doi: 10.3390/metabo14090483 (PMC11434292; doi:10.3390/metabo14090483)
Supplement: Supplementary file 1 [file metabolites-14-00483-s001.zip › Park et al_Supplementary materials.pdf]

Supplementary Materials

Table S1. Model performance metrics in DIO mice

Machine learning model	AUC	CA	F1	Precision	Recall	MCC
Neural Network	0.985	0.912	0.912	0.921	0.912	0.834
Random Forest	0.981	0.902	0.902	0.905	0.902	0.807
Logistic Regression	0.952	0.863	0.862	0.865	0.863	0.728
SVM	0.918	0.925	0.925	0.926	0.925	0.851
Gradient Boosting	0.828	0.828	0.827	0.829	0.828	0.657
kNN	0.717	0.688	0.686	0.691	0.688	0.378
AdaBoost	0.705	0.705	0.705	0.705	0.705	0.410

AUC, area under the curve; CA, classification accuracy; MCC, Matthews correlation coefficient;
F1, 2 × (Precision × Recall / Precision + Recall)

Table S2. Model performance metrics in T2DM patients

Machine learning model	AUC	CA	F1	Precision	Recall	MCC
Gradient Boosting	0.878	0.853	0.853	0.853	0.853	0.706
AdaBoost	0.819	0.819	0.819	0.821	0.819	0.640
Logistic Regression	0.787	0.716	0.716	0.717	0.716	0.433
Random Forest	0.736	0.726	0.726	0.727	0.726	0.453
kNN	0.611	0.572	0.570	0.573	0.572	0.145
Neural Network	0.472	0.493	0.486	0.493	0.493	-0.014
SVM	0.427	0.610	0.586	0.644	0.610	0.251

AUC, area under the curve; CA, classification accuracy; MCC, Matthews correlation coefficient;
F1, 2 × (Precision × Recall / Precision + Recall)

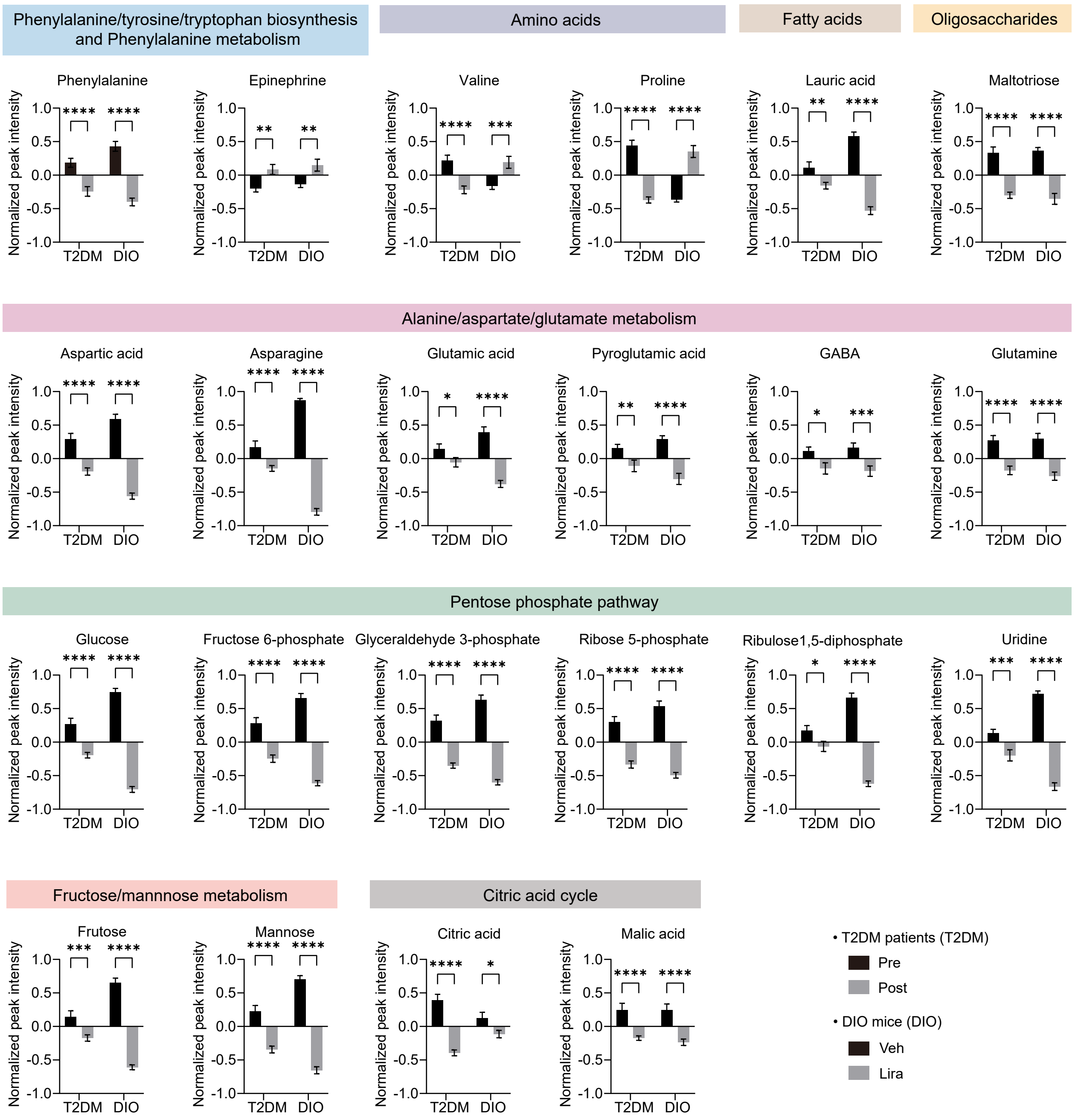

Figure S1. Plasma metabolites altered by liraglutide in T2DM patients and DIO mice. Bar graphs depict metabolites significantly altered by liraglutide, categorized by metabolic pathways. Statistical significance was determined by two-way ANOVA followed by Fisher's LSD test. $*p < 0.05$, $**p < 0.01$, $***p < 0.001$, $****p < 0.0001$; ns, not significant. Data are presented as mean ± SEM. Asterisks are only displayed in the comparison groups within T2DM patients (Pre vs. Post) and DIO mice (Veh vs Lira).

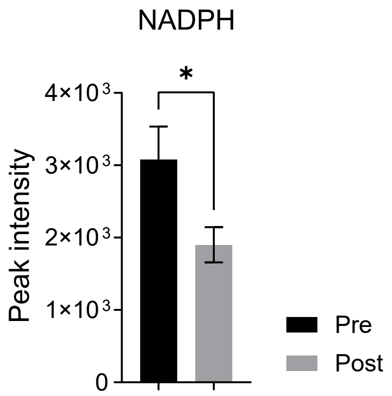

Figure S2. Effect of liraglutide on plasma NADPH level in T2DM patients. Statistical significance was determined by a two-tailed unpaired Student's *t*-test. *p <0.05. Data are presented as mean ± SEM.
